# Ice-Assisted Porous Poly(ionic liquid)/MXene Composite Membranes for Solar Steam Generation

**DOI:** 10.1021/acsami.3c15551

**Published:** 2023-11-20

**Authors:** Atefeh Khorsand Kheirabad, Helena K. J. Friedrich, Jian Chang, Miao Zhang, Andre Gröschel, Jiayin Yuan

**Affiliations:** † Department of Materials and Environmental Chemistry (MMK), Stockholm University, 10691 Stockholm, Sweden; ‡ Institute for Physical Chemistry and Center for Soft Nanoscience (SoN), University of Munster, 48149 Munster, Germany

**Keywords:** poly(ionic liquid), ice-assisted fabrication, MXene, porous polyelectrolyte composite membrane, photothermal conversion

## Abstract

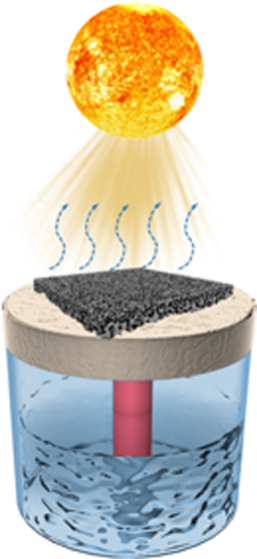

Controlled synthesis of polymer-based porous membranes via innovative methods is of considerable interest, yet it remains a challenge. Herein, we established a general approach to fabricate porous polyelectrolyte composite membranes (PPCMs) from poly(ionic liquid) (PIL) and MXene via an ice-assisted method. This process enabled the formation of a uniformly distributed macroporous structure within the membrane. The unique characteristics of the as-produced composite membranes display significant light-to-heat conversion and excellent performance for solar-driven water vapor generation. This facile synthetic strategy breaks new ground for developing composite porous membranes as high-performance solar steam generators for clean water production.

## Introduction

Recently, porous polyelectrolyte membranes (PPMs) have become increasingly appealing in both academia and industry due to the combination of their specific characteristics of charged nature and variation in pore size distributions,^1^ and their potential in a wide range of applications in energy devices,^2^ sensors,^3^ separation technology,^4^ and batteries,^5^ just to name a few.^6^ The construction of PPMs and their hybrids and composites is mostly based on traditional methods including layer-by-layer assembly,^7^ hard templating strategies,^8^ electrostatic interactions or covalent cross-linking for self-assembly of block copolymers and multicomponent polymeric materials.^9,10^ However, these synthetic techniques are intensive in time and labor work. In this context, there are continuous efforts to explore alternative facile approaches to prepare PPMs, in particular, freestanding ones.

Poly(ionic liquid)s (PILs) with ionic liquid (IL)-derived species in their repeating units have recently emerged as a subclass of polyelectrolytes with the capability to provide a multifunctional material platform in the membrane field.^11−13^ The unique features of PILs including their variations in hydrophobicity and solubility in organic solvents provide an opportunity to compound with other organic and inorganic materials. Although a fabrication method based on electrostatic cross-linking of a hydrophobic cationic PIL with a weak polyanion was developed previously to spawn a myriad of functional PPMs for demonstrative applications,^14^ PPMs with more controlled structure can be exploited to expand their applications specifically in the field of energy and environment as solar-driven materials with focus on pressing global issues, e.g., generation of clean water.^15^ Thus, based on the criteria of an ideal interfacial solar evaporator such as strong solar-to-thermal conversion, high thermal insulation on bulk water, porous structure to transfer water supply, and escape of produced vapors, the combination of PIL with other functional materials would provide a new dimension to broaden the actual application scope of PPCMs, which was previously less explored.^16−18^

The ice-templating or freeze-casting processes have been utilized enormously to fabricate a wide variety of porous materials with great potential in several engineering fields^19−21^ including energy,^22,23^ biomedical,^24^ catalysis,^25^ and separation.^26^ In comparison to other processes, methods using the assistance of freezing the component solution exhibit several unique features, i.e., a variety of porous structures based on the choice of the solvent suspension formulation (*e.g.*, solid content and additives) and the solidification conditions (*e.g.*, temperature gradient and cooling rate). In addition, a diverse range of architectures such as thin filaments, structural foams, membranes, and microspheres can be accessed by such methods. Thus, ice-templated materials with comprehensive porous structures have attracted significant interest due to the possibility of modulating the nucleation, growth, shapes, sizes, and hierarchy of ice templates, which leads to the emergence of polymer composite materials with multiple features including electrical, piezoelectric, thermal, mechanical, and catalytic properties. Certainly, freeze-casting methods can endow a variety of hybrid and composite materials with versatile and integrated functions.^27,28^ Specifically, a solution of PIL can be subjected to the ice-templating method and produce a porous morphology. The pores in this structure can be tailored in size and distribution by adjusting the freezing conditions and/or in combination with other mechanisms such as phase separation of a hydrophobic cationic PIL in water, electrostatic complexation due to cross-linking with a weak acid.^14,29^

Lately, MXenes as a family of two-dimensional (2D) transition metal carbides, carbonitrides and nitrides,^30^ have been assembled into porous materials by freeze-casting methods.^31−33^ Pioneering studies were reported on the promising potential of MXene-based photothermal materials when used as solar absorbers in solar desalination applications.^34^ As for photothermal materials, MXenes are rather limited by inherently undesirable characteristics, such as high thermal conductivity, moderate broadband light reflection, and its tendency to be easily oxidized.^35−38^ These unfavorable aspects restrict the photothermal conversion efficiency of MXene-based composite materials for solar vapor generation, and considerable efforts have been directed to address these concerns.^39−41^

In this study, we established a fabrication method to prepare ice-assisted PPMs with task-specific architectures. Briefly, a cationic PIL was dissolved in dimethyl sulfoxide (DMSO), and then the solution was frozen quickly and immersed in an aqueous bath containing a phosphonic acid compound. The frozen solution slowly melted in water, accompanied by the simultaneous cross-linking of the cationic PIL chain by the phosphonic acids. At the same time, a phase separation process occurred due to the slow diffusion of water molecules through the frozen solution of the hydrophobic PIL and the parallel solvent exchange process with DMSO molecules during slow melting in an aqueous bath. These two simultaneous processes initiated the creation of a macroporous architecture in the membrane. In the following, this method was employed to produce PPCM from the composition of a cationic PIL and titanium carbide type of MXene. This porous PIL/MXene composite membrane was successfully applied as a solar absorber, which performed at a high evaporation rate while maintaining its persistency and stability over time.

## Experimental Section

### Materials

All chemicals were utilized as received from the chemical suppliers without any further purification. MAX phase powder (Ti_3_AlC_2_, ≥ 99%, 400 mesh) was received from Laizhou Kai Kai Ceramics Materials Co., Ltd. Hydrochloric acid (HCl, 37%) was purchased from VWR International. Phytic acid solution (PA, 50% (w/w) in water), diethylentriaminepentakis(methylphosphonic acid) solution in water (≅50% T), lithium fluoride (LiF, ≥ 99.98% trace metals basis), lithium bis(trifluoromethylsulfonyl)imide (LiTFSI, 99%), 1-vinylimidazole (99%), azodiisobutyronitrile (AIBN, 98%), and bromoacetonitrile (97%) were obtained from Sigma-Aldrich. Solvents were of analytical grade.

### Characterizations

The imaging of the morphology of samples was performed by scanning electron microscopy (SEM) performed on a JEOL 7000 microscope. The operated accelerating voltage was 2 kV. Before testing, samples were coated with a thin layer of gold. The morphology and size of MXene nanolayers were studied using transmission electron microscopy (TEM) which was performed on a JEOL JEM- 2100F instrument under an accelerating voltage of 100 kV. The thickness of MXene nanosheets was measured via atomic force microscopy (AFM) which was conducted utilizing a Nanoscope V (Veeco Instruments) in the tapping mode. X-ray diffraction (XRD) patterns were recorded using an X-ray diffractometer (PANalytical X’Pert Pro) applying Cu Kα radiation (λ = 1.5418 Å) at room temperature with a scanning speed of 5° min^–1^ in the range of diffraction angle 2θ of 5° to 80° on the reflection mode. Transmission and reflection spectra of membranes in the dry state were measured using an ultraviolet–visible–near-infrared spectrophotometer (Agilent Cary 5000 UV–vis–NIR). The transmittance spectra of membranes were recorded in the range of 200–2500 nm with air as a background. The reflectance spectra of produced membranes were supported by an integrating sphere attachment and a specific fluorine-based polymer (PTFE) as a reference. Both infrared (IR) camera and thermocouple were utilized to examine the photothermal conversion performance of membranes. An IR camera (Testo 872) was used to collect thermal images, and K-type thermocouple (KAIPUSEN) was used to record the temperature change of membranes in a specific period of time at ambient condition. During the measurement, solar irradiation was provided by using an artificial solar simulator (MiniSol LED Solar Simulator, Newport). Proton nuclear magnetic resonance (^1^H NMR) spectra were recorded at room temperature by using a Bruker DPX-400 spectrometer operating at 400 MHz. For the measurement, samples were dissolved in DMSO-*d*_6_.

### Synthesis of Poly(1-cyanomethyl-3-vinylimidazolium Bis(trifluoromethylsulfonyl)imide) (PCMVImTFSI)

1-Vinylimidazole (23.5 g, 0.25 mol) and bromoacetonitrile (30 g, 0.25 mol) were mixed and dissolved in diethyl ether (55 mL). The solution was stirred overnight at room temperature. The reaction mixture was then filtered off, and the solid was washed three times with diethyl ether and dried in a vacuum oven at 40 °C overnight. In the next step, the obtained white powder of 1-cyanomethyl-3-vinylimidazolium bromide (10 g) and AIBN (200 mg) were added to DMSO (100 mL) and placed in a 250 mL Schlenk flask. The reaction mixture was deoxygenated three times by a freeze–pump–thaw procedure. The temperature of the reaction mixture was maintained at 70 °C overnight. For purification of the polymer, the reaction mixture was added to tetrahydrofuran (THF) (2 L) dropwise. The precipitate was filtered off, washed with THF at least three times, and then dried at 70 °C under vacuum overnight. In the following, the dried, slightly yellow powder of poly(1-cyanomethyl-3-vinylimidazolium bromide) (10 g) was dissolved in 200 mL of deionized water. Bis(trifluoromethylsulfonyl)imide lithium salt (12.7 g) was dissolved in deionized water and then added drop by drop to an aqueous solution of poly(1-cyanomethyl-3-vinylimidazolium bromide). This reaction mixture was stirred for at least 2 h up to overnight. The precipitate was then filtered off, washed several times with deionized water, and dried under a vacuum at 70 °C overnight. The chemical structures of the obtained compounds were examined and confirmed by ^1^H NMR spectrum as shown in Figure S1.

### Synthesis of MXene Nanosheets

1.5 g of MAX powder with high purity (1.5 g, equivalent to 12 M) was added carefully into a mixture of LiF (2.4 g) and HCl (30 mL, 9M) under persistent stirring in a period of 10 min and constant cooling using an ice–water bath. This reaction mixture was kept under stirring for 36 h at room temperature. In the following, the multilayer Ti_3_AlC_2_ and unetched MAX particles were washed and centrifuged (at 10,000 rpm for 1 h for 7 or 8 cycles) with deionized water until reaching a pH value of ca. 6 in the supernatant fluid. The suspension of Ti_3_C_2_T_*x*_ MXene sheets was produced by the exfoliation process of the resultant slurry under sonication for 10 min under a nitrogen flow. The unexfoliated MXenes were separated via centrifugation at 3000 rpm. The concentration of MXene suspension was calculated by measuring the weight of dried MXene nanosheets after freeze-drying 2 mL suspensions. For this study, water was exchanged with DMSO using centrifugation (at 11,000 rpm for 1 h for 3–4 cycles), and MXene nanosheets were redispersed in DMSO based on the required concentration.

### Calculations

The efficiency of solar evaporation for each membrane was calculated by eq 1:
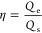
1where *Q*_s_ is the value of power density for the solar irradiation (1000 W m^–2^) and *Q*_e_ is the power of water evaporation, which is determined using eq 2:
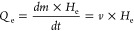
2where *m* is the mass of evaporated water, *H*_e_ is the latent heat of water evaporation (2444.0 kJ kg^–1^ at 25 °C and 2357.7 kJ kg^–1^ at 60 °C), *t* is the duration time of the experiment, and *v* is the evaporation rate of water of each membrane, calculated using eq 3:
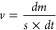
3where *m* is the mass of evaporated water, *s* is the surface area of the used membrane, and *t* is the time period of test.

## Results and Discussion

**Scheme 1:**
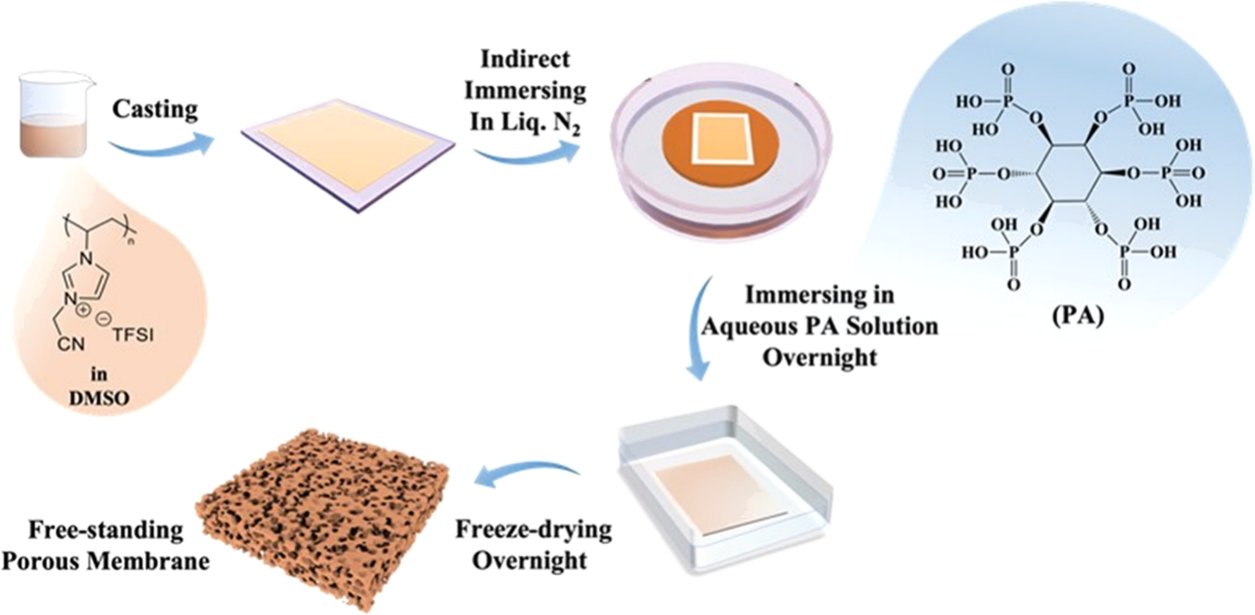
Schematic of Experimental Procedure Based on an Ice-Assisting Method to Prepare the Porous PIL Membrane

Scheme 1 displays the schematic procedure to fabricate PIL-based membranes. In the first step, a cationic water-insoluble PIL, poly(1-cyanomethyl-3-vinylimidazolium bis(trifluoromethylsulfonyl)imide) (termed PCMVImTFSI) was dissolved homogeneously in DMSO solvent. Next, the slightly yellowish solution was poured into a plastic container and instantly frozen using liquid nitrogen. The frozen PIL solution was then immersed into an aqueous phosphonic acid solution at room temperature and remained for overnight. Phytic acid (termed PA) was used as one type of phosphonic acid. Then, the formed opaque membrane was washed with distilled water several times to remove the remaining excessive acid. Finally, the prepared membrane in a wet state was freeze-dried for at least 24 h, resulting in a freestanding porous membrane, which could easily be detached from the substrate.

**Figure 1:**
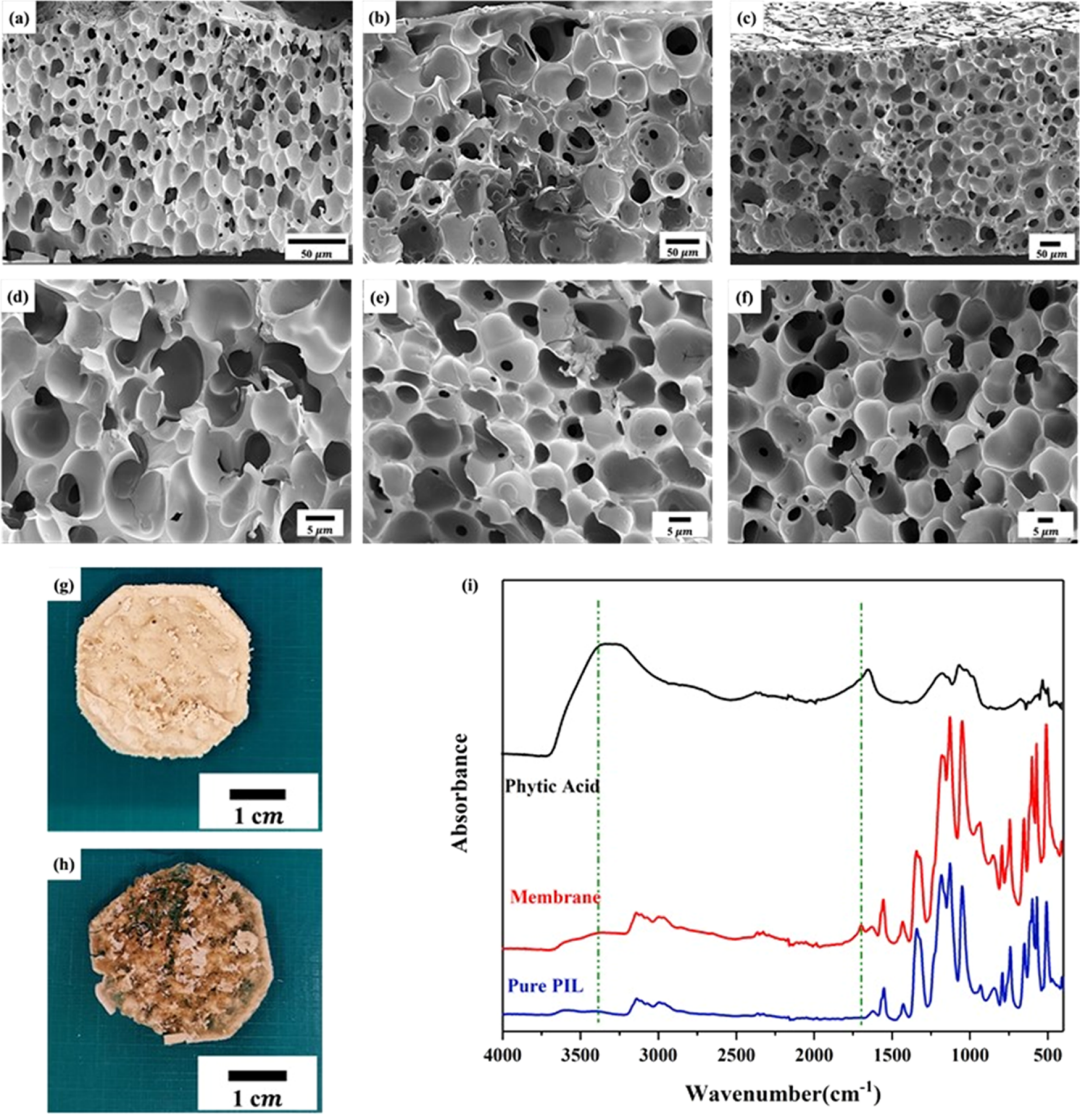
Low- and high-magnification cross-sectional SEM images of porous PIL membranes prepared at a PIL concentration of (a, d) 100 mg/mL, (b, e) 150 mg/mL, and (c, f) 200 mg/mL in DMSO. Digital images of dried membranes prepared with (g) and without (h) PA in an aqueous bath, respectively. (i) FTIR spectra of phytic acid (black), porous membrane (red), and pristine PIL (blue).

In order to validate the relevance of each effective parameter during membrane formation, a series of controlled experiments were conducted. Since the first step of this method consists of preparing the PIL solution in DMSO, three different concentrations of 100, 150, and 200 mg/mL were employed to study their effect on the membrane morphology. After pouring each solution into a plastic mold, the freezing step was induced indirectly by connecting the bottom of the container in contact with a cold glass stage, which was placed into a liquid nitrogen bath to control the growth of ice crystals and prevent direct contact of PIL solution with liquid nitrogen. During the following step, each frozen sample was soaked inside an aqueous PA solution, which can be deprotonated in water to electrostatically cross-link the cationic PIL into a porous membrane. When the aqueous PA solution encounters the surface of frozen PIL/DMSO, it diffuses into the polymer chains that are released from the solid mixture. This triggers *in situ* ionic complexation of PA with the released PIL chains to build up the ionically cross-linked network. In parallel, a phase separation event due to the diffusion of water molecules into DMSO to contact the hydrophobic PIL polymer chains occurs, which leads to the formation of macropores within the membrane. It should be noted that the concentration of the aqueous acid solution was determined based on an equivalent molar ratio between imidazolium units of the cationic PIL and the hydroxyl units of PA compound. Cross-sectional scanning electron microscopy (SEM) images at low magnification provided in Figure 1a–c represent the general overview of the created membranes using this method. The thickness of each membrane was determined to be 206.5 ± 10.3, 383.4 ± 7.2, and 509.7 ± 20.9 μm when utilizing the PIL concentration of 100, 150, and 200 mg/mL, respectively. Furthermore, high-magnification cross-sectional SEM images (Figure 1d–f) revealed the spherical morphology of created pores with an average pore size of 6.4 ± 2.9, 9.3 ± 3.8, and 12.7 ± 4.7 μm for membranes prepared at PIL concentration of 100, 150, and 200 mg/mL, respectively. As it can be clearly observed, the thickness of the membrane gradually increased when a higher concentration of PIL solution was applied. From a kinetic point of view, the diffusion of water and PA molecules into the frozen mixture was delayed because of the increased PIL concentration and resulted in larger pores. The pore size distribution of each membrane is provided in Figure S2.

In a control experiment, we soaked the frozen PIL/DMSO solid solution in neutral water without PA, which did not result in intact membranes due to weak mechanical properties of the formed film. Digital images of obtained membranes with and without PA in water are shown in Figure 1g,1h, respectively. This comparison clearly demonstrates that the presence of PA promotes the formation of a stable polyelectrolyte membrane. The Fourier transform IR spectrum (FTIR) provided in Figure 1i shows absorption bands at 1700 and 3700–3300 cm^–1^ attributed to P=O stretching and −OH vibration in PA, respectively, confirming the presence of hydroxyl groups (OH) of PA in the membrane. Furthermore, elemental analysis of a dried membrane showed the amount of phosphorus (P) to be 1.77 wt %.

Since the presence of PA is crucial to form a stable freestanding porous membrane, we varied the PA content and prepared an aqueous solution of 4 g of PA dissolved in 50 mL of water. To note that the amount of PA was calculated based on 1/1 equiv molar ratio between hydroxyl groups of PA and imidazolium units of cationic PIL (OH/IL). Furthermore, other molar ratios for the preparation of PA aqueous solution between 1/1 and 1/20 were also employed. The respective SEM images of the formed membranes (Figure S3) exhibit similar morphologies with similar pore size. However, further decreasing the OH/IL molar ratio resulted in less stable membranes with weaker cross-links and the obtained membranes were hard to detach from the plastics substrate. Other types of phosphonic acid compounds can also be used instead of PA in this method. For instance, diethylentriaminepentakis(methylphosphonic acid) (termed TA) was used to form a stable porous membrane, and the digital image of formed membrane is provided in Figure S4. The thickness of membranes formed by utilizing TA aqueous solution with 1/1 equiv molar ratio of OH/IL, and soaking frozen PIL in TA aqueous solutions was measured (Figure S5) to be 154.7 ± 10.1, 246.3 ± 2.9, and 305.5 ± 3.4 μm for membranes prepared at a PIL concentration at 100, 150, and 200 mg/mL, respectively.

**Figure 2:**
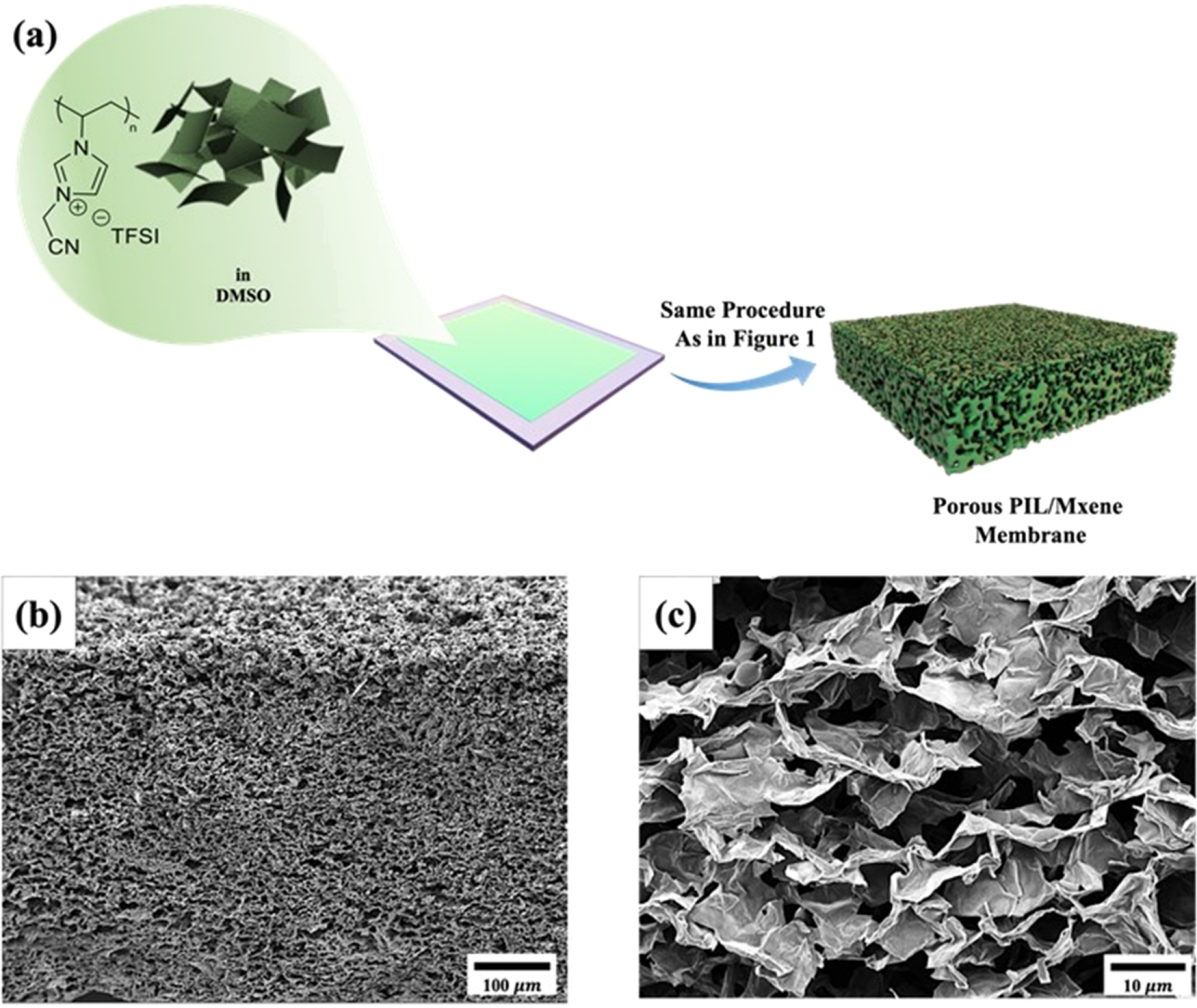
(a) Schematic of experimental procedure to prepare composite PIL/MXene porous membrane using the same ice-assisted method to PPM. (b, c) Low- and high-magnification SEM images from the cross-section of a prepared composite membrane using 150 mg of PIL and 10 mg of MXene dispersed homogeneously in DMSO.

The versatility of this ice-assisted method was further validated by the preparation of a composite porous membrane by adding MXene nanosheets, here Ti_3_C_2_T_*x*_, as a 2D additive. MXene nanosheets were well-dispersed in DMSO and added into the clear solution of PIL in DMSO under vigorous stirring to reach a homogeneous dispersion without any optical aggregates. The formed membrane via the same procedure using this solution was termed PIL/MXene (Figure 2a). Here, an amount of 10 mg of pristine MXene was chosen to be mixed with 150 mg of the PIL solution. Cross-sectional SEM images showed a three-dimensional (3D) interconnected porous architecture for the PIL/MXene membrane with an average thickness of 467.5 ± 6.1 μm (Figure 2b). The increase in membrane thickness compared to that of the pristine PIL membrane (383.4 ± 7.2 μm) obviously originates from the MXene additives. High-magnification cross-sectional SEM images of the composite PIL/MXene membrane in Figure 2c shows its morphology with an average pore size of 3.9 ± 1.2 μm. The comparison with an average pore size of the reference PIL-based MXene-free porous membranes (9.3 ± 3.8 μm) indicates that the MXene nanolayers had a significant effect on reducing the average pores size.

The synthetic method to prepare Ti_3_C_2_T_*x*_ MXene nanosheets is presented in the Experimental Section and briefly described here. In the first step, atomic layers of aluminum from the bulk MAX phase (Ti_3_AlC_2_) were etched away by concurrent intercalation of Li^+^ ions in a mixture of LiF and HCl. The as-synthesized MXene nanosheets were analyzed using transmission electron microscopy (TEM, Figure S6) and atomic force microscopy (AFM, Figure S7). The synthesized nanosheets featured a lateral size of 0.8–4 μm and a height of 0.7–1.8 nm with a smooth surface. The theoretical thickness for MXene monolayers with a surface bound water layer is approximately 1.0 nm,^32,33,36^ suggesting that the as-prepared MXene nanosheets were monolayers. The X-ray diffraction (XRD) patterns (Figure S8) obtained from the freeze-dried MXene aqueous solutions confirm the successful transformation of the MAX phase into 2D MXene nanolayers; the (104) plane at 39° disappeared after successful exfoliation. The diffraction peak at 6.5° of the MXene nanosheets is assigned to the (002) lattice plane, which shows a much higher intensity in the X-ray diffraction diagram with a shift to a lower degree in comparison to those of the MAX phase, which confirms the regular stacking of MXene nanosheets. All of the above-mentioned characterization results verified the delamination of MXene nanosheets via the etching treatment of the bulk MAX.^37^ In order to receive a more profound insight into the lamellar structure of MXene nanolayers of the composite PIL/MXene porous membranes, the XRD patterns of the pristine MXene and PIL/MXene membranes were compared, and the *d*-spacing (interlayer spacing) was measured (Figure S9). The sharp (002) peak located at 5.64° indicates a *d*-spacing value of 1.57 nm for the pristine MXene membrane, consistent with recent literature.^42−44^ PIL/MXene with a higher *d*-spacing value of 1.61 nm was calculated, indicating that the PIL was intercalated into the MXene multilayers.

**Figure 3:**
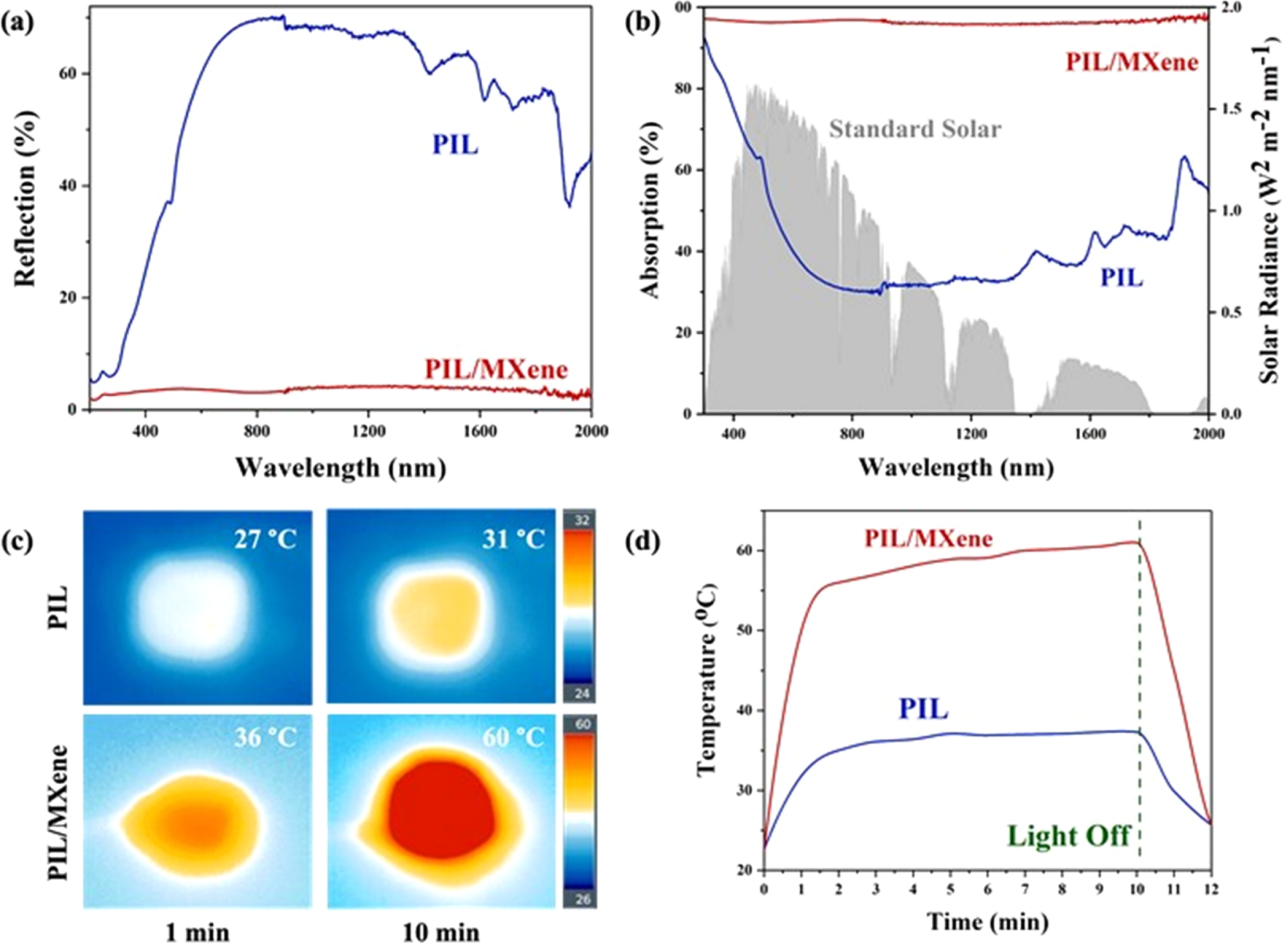
UV–Vis–NIR spectra of (a) reflection and (b) absorption, (c) infrared images, and (d) surface temperature over time under one-sun irradiation for PIL-based and PIL/MXene membranes.

Optical absorption is a crucial characteristic directly impacting the solar vapor generation. To quantify the optical absorption properties of the MXene-based composite solar absorbers, optical absorption, reflection, and transmittance spectra were recorded using an ultraviolet–visible–near-infrared (UV–vis–NIR) spectrophotometer equipped with an integrating sphere. While both the PIL and PIL/MXene porous membranes exhibited relatively low transmittance throughout the entire solar spectrum (Figure S10), the incident light irradiated on the PIL/MXene membrane exhibited a significantly lower reflection (Figure 3a). The uniform and finely structured surface of the porous PIL/MXene membrane further decreases the light reflection, which is beneficial for solar absorption. Correspondingly, the PIL/MXene composite membrane had an average absorption of over 95% in the visible to near-infrared range, which is higher than the average absorption of pure PIL membrane (between 30 and 80%, Figure 3b). Based on the normalized spectral solar irradiance density, the absorbed solar energy of the PIL/MXene composite membrane was calculated to be 97.5%, much higher than that of the PIL membrane. Upon one-sun irradiation (1 kW m^–2^), the surface temperature of the PIL/MXene membrane increased from 30 to 62 °C in a dry state within 10 min. The final temperature is twice that of the PIL membrane (∼31 °C) under identical irradiation conditions (Figure 3c). These results prove the improved solar-thermal conversion capability of the PIL/MXene over the pure PIL membrane, most likely due to the increased reflection of light on the black surface and the smaller pore channels within the PIL/MXene membrane. In addition, infrared images recorded via an IR thermal camera under one-sun illumination in the equilibrium state (Figure 3d) disclosed the positive correlation of the surface temperature with the MXene content within the membrane. These results indicate the effective light absorption of the composite membrane that is beneficial for solar–thermal steaming of water.

**Figure 4:**
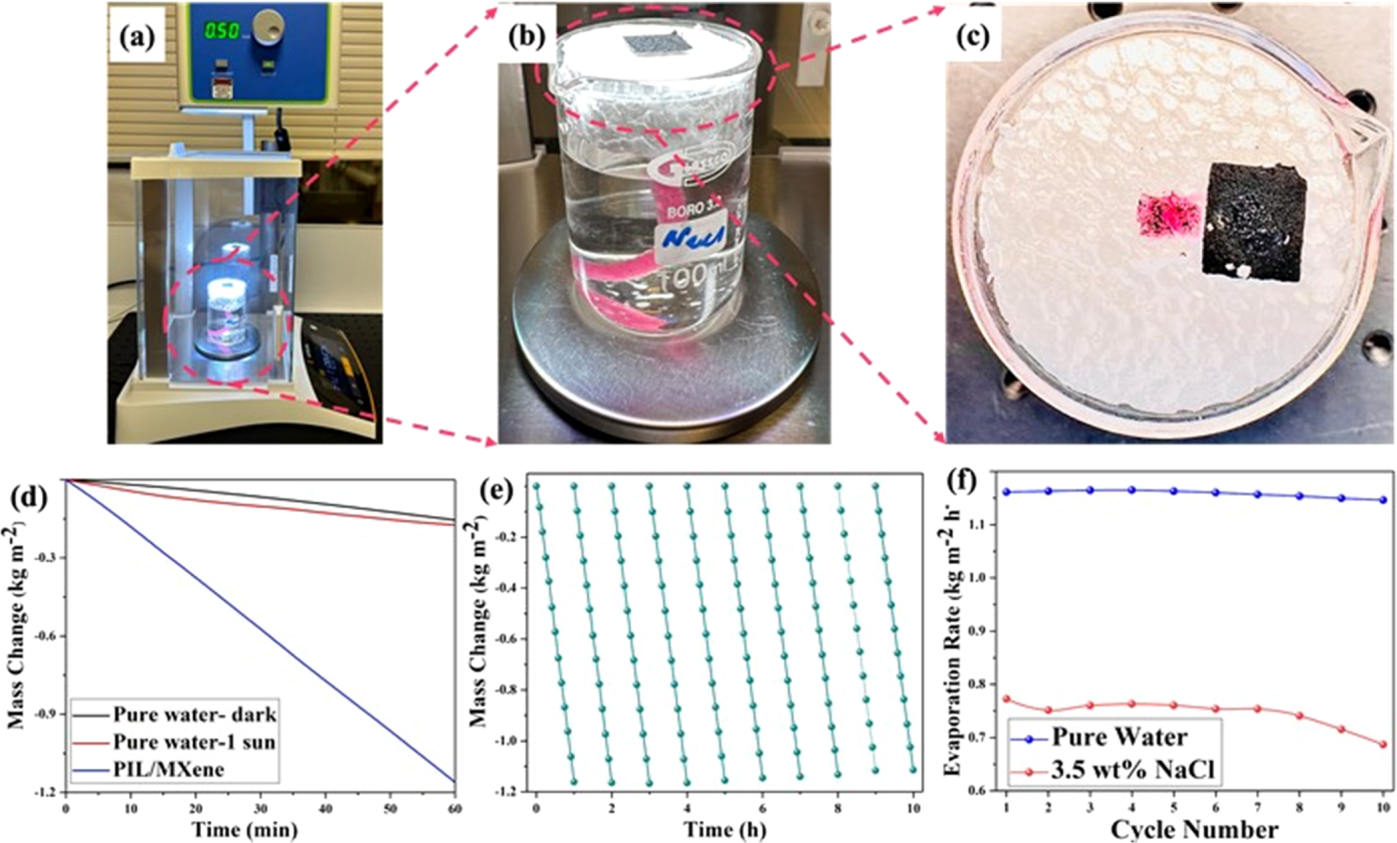
Digital images of (a) the overview of solar-driven water evaporation setup, (b) isolated water evaporation using a beaker, thermal insulator, and PIL/MXene porous membrane, and (c) close view of the accumulated salt on PIL/MXene membrane after running over time. (d) Mass change of water under 1 kW m^–2^ irradiation using PIL and PIL/MXene porous membranes as solar absorber. (e) Performance stability test of PIL/MXene porous membrane during 10 h of operation. (f) Solar–thermal evaporation rate of distilled water and salty water using PIL/MXene porous membrane under one-sun irradiation.

Figure 4a shows a digital image of the solar-driven steam generation setup to study the application of this composite porous membrane. A square PIL/MXene porous membrane with a length of 1.2 cm was placed on the top of a 1.8 cm-thick polystyrene (PS) foam as a thermal isolator to minimize the heat dissipation from the photothermal porous membrane to the underneath bulk water. A cotton cloth strip was inserted through a hole inside the PS foam and utilized as a capillary water channel to pull water from the bulk water body to the surface of the porous membrane. This capillary water channel provides water to the porous membrane and wets the entire membrane continuously due to the porous structure of the membrane and the hydrophilicity of the MXene nanosheets. This setup was placed on a digital scale connected to a computer to monitor the real-time change of water evaporation under constant 1 kW m^–2^ irradiation (Figure 4b,c allows a closer look into details of this design). Figure 4d presents details about mass change as a function of time, where a water evaporation rate was calculated from the slope of the related curve. The mass of water decreases linearly with increasing irradiation time, and the evaporation rate of bulk water is 1.2 kg m^–2^ h^–1^ when using the PIL/MXene membrane under one-sun irradiation, which is 6 times higher than the bulk water evaporation rate (0.2 kg m^–2^ h^–1^) without using the composite membrane. Based on the calculated water evaporation rate, the energy conversion efficiency (eq 3) was then calculated to be 92% for the porous PIL/MXene membrane. Besides, the PIL/MXene membrane shows stable performance (1.16–1.2 kg m^–2^ h^–1^) and intact structure during the 10 h of continuous solar–thermal evaporation (Figure 4e). In order to further demonstrate the feasibility of the real-life implementation of this design, the evaporation rate of this membrane was recorded using salty water (3.5 wt % of NaCl) for 10 sequential cycles and compared with pure water (Figure 4f). Although the evaporation rate of salty water (ca. 0.7 kg m^–2^ h^–1^) was lower than that of pure water (ca. 1.2 kg m^–2^ h^–1^), the PIL/MXene membrane structure remained stable and can, therefore, be used to harvest salt from water. The enclosed digital image in Figure 4c shows the collected salt under the PIL/MXene membrane after running overnight continuously. The total rate of vapor production during this test period was calculated to ca. 6.5 kg m^–2^.

## Conclusions

In conclusion, we established a fabrication method for the preparation of composite porous membranes containing a cationic hydrophobic PIL (PCVImTFSI) and MXene nanosheets as additives using an ice-assisted method via ionic cross-linking with phosphonic acid in water. The formation of a porous polyelectrolyte membrane architecture with MXene nanolayers paves the way for engineering task-specific membranes possessing macropores with tunable thickness and an inner pore structure. The as-synthesized PIL/MXene porous membranes exhibited high broadband light absorption, low light transmittance, efficient light-to-heat conversion, high water evaporation rate, persistent structure, and stable performance under 10 continuous test cycles. Furthermore, the PIL/MXene composite membrane was tested using salty water (simulated seawater), which showed stable performance and structural stability when running the experiment constantly overnight. We envision that this preparative strategy opens a new avenue for the development of porous membrane-based solar absorbers and salt harvesting in energy fields.

## Supporting Information Available

Additional characterization details, including ^1^H NMR spectra for all compounds, additional cross-sectional SEM images of membranes, TEM and AFM images of MXene nanosheets, XRD patterns of MAX and MXene and composite membranes, UV–vis–NIR spectra of transmittance of membranes, and the comparison evaporation rate and efficiency of this work with other composite materials (PDF)

## Abbreviations

PIL: poly(ionic liquid)
PPM: porous polyelectrolyte membrane
PPCM: porous composite polyelectrolyte membrane
SEM: scanning electron microscopy
TEM: transmission electron microscopy
AFM: atomic force microscopy
XRD: X-ray diffraction
DMSO: dimethyl sulfoxide
THF: tetrahydrofuran
