# Supplementary material for: Ice-Assisted Porous Poly(ionic liquid)/MXene Composite Membranes for Solar Steam Generation
Source: ACS Appl Mater Interfaces. 2023 Nov 20;15(48):56347–55. doi: 10.1021/acsami.3c15551 (PMC10711704; doi:10.1021/acsami.3c15551)
Supplement: Supplementary file 1 — am3c15551_si_001.pdf [file am3c15551_si_001.pdf]

Supporting Information

Ice-Assisted Porous Poly(ionic liquid)/MXene Composite Membranes for Solar Steam Generation

Atefeh Khorsand Kheirabad[1], *Helena K. J. Friedrich*[1,2], *Jian Chang*[1], *Miao Zhang*[1], *Andre Gröschel*[2,*] *Jiayin Yuan*[1,*]

[1]Department of Materials and Environmental Chemistry (MMK), Stockholm University, 10691 Stockholm, Sweden.

[2] Institute for Physical Chemistry and Center for Soft Nanoscience (SoN), University of Munster, 48149 Munster, Germany.

E-Mail: andre.groeschel@uni-muenster.de; jiayin.yuan@mmk.su.se

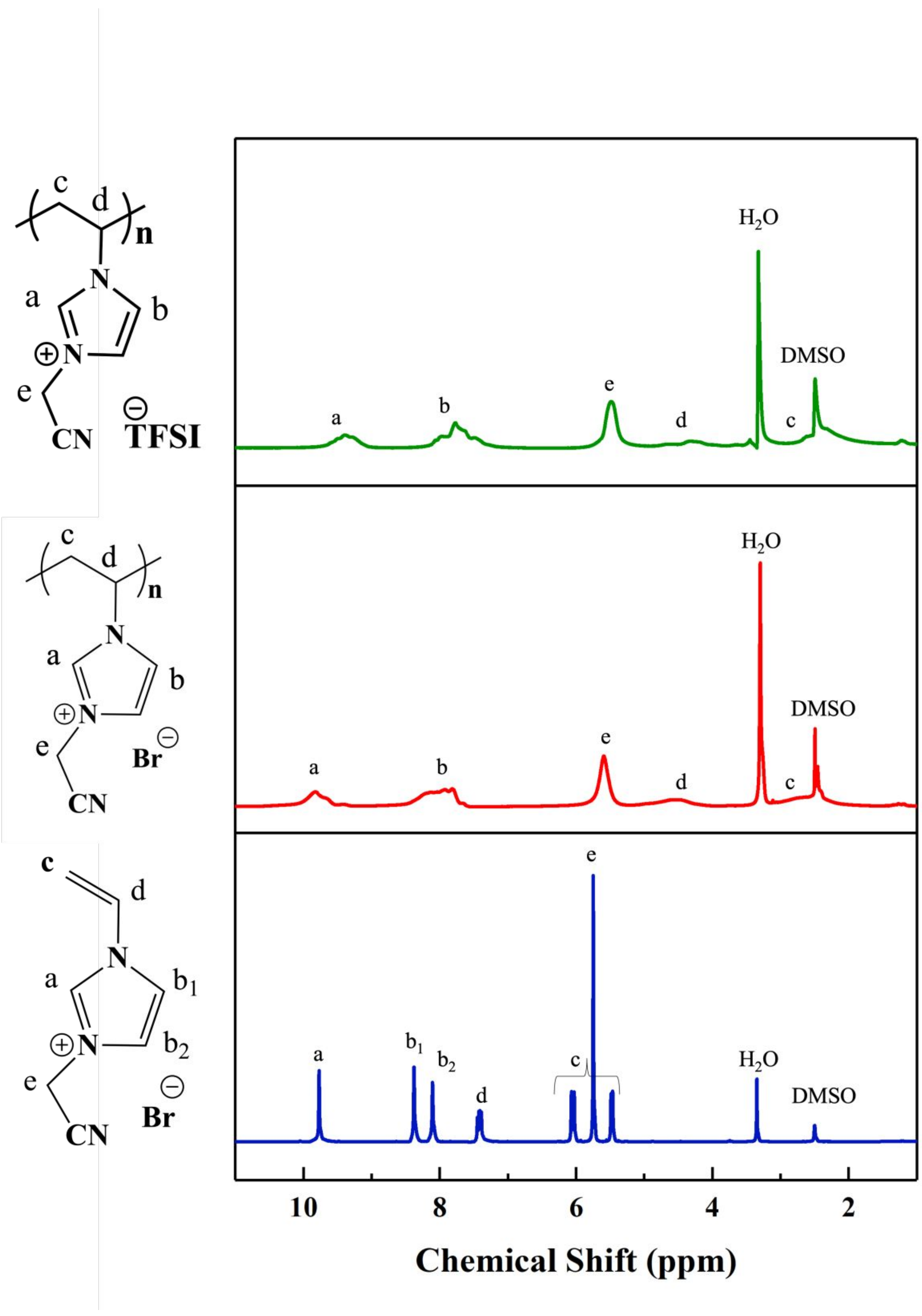

c
d
n
N
a
b
N
e
CN
TFSI
H2O
DMSO
a
b
e
d
c
Br
e
d
c
b1
b2
10
8
6
4
2
Chemical Shift (ppm)

Figure S1. 1H-NMR spectrum of 1-cyanomethyl-3-vinylimidazolium bromide, poly(1-cyanomethyl-3-vinylimidazolium bromide, and poly(1-cyanomethyl-3-vinylimidazolium bis(trifluoromethylsulfonyl)imide) in DMSO-d6.

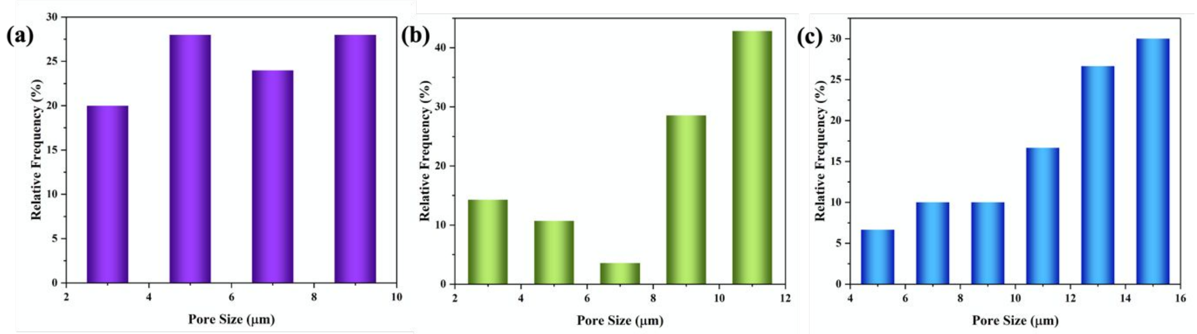

Figure S2. Pore size distribution of porous PIL membranes prepared with (a) 100 mg/ml, (b) 150 mg/ml and (c) 200 mg/ml amount of PIL dissolved in DMSO solution.

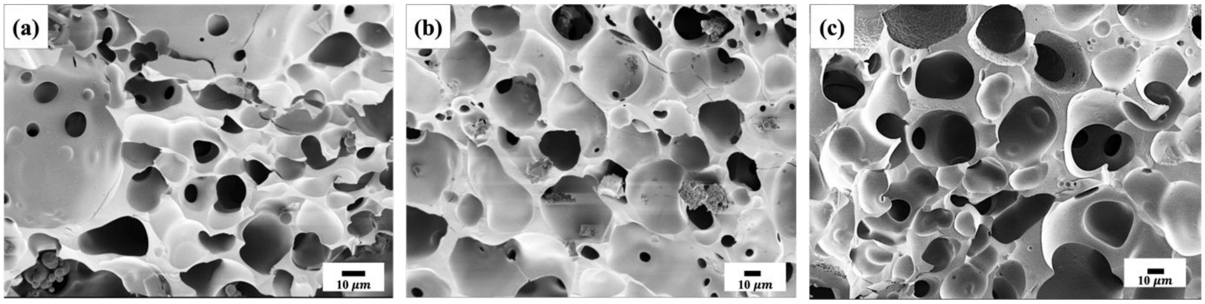

Figure S3. Cross-sectional SEM images of porous PIL membranes prepared with fixed amount of PIL (200 mg/ml) dissolved in DMSO solution and (a) 6/1, (b) 10/1 and (c) 20/1 equivalent molar ratio of OH/IL between hydroxyl groups of PA and imidazolium units.

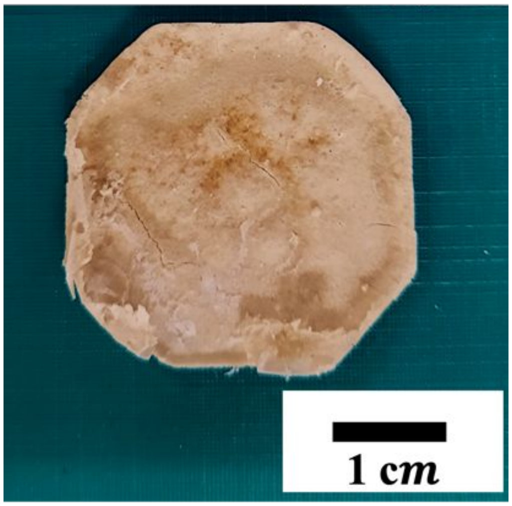

Figure S4. Digital image of a free-standing porous membrane prepared using TA as a phosphonic acid source.

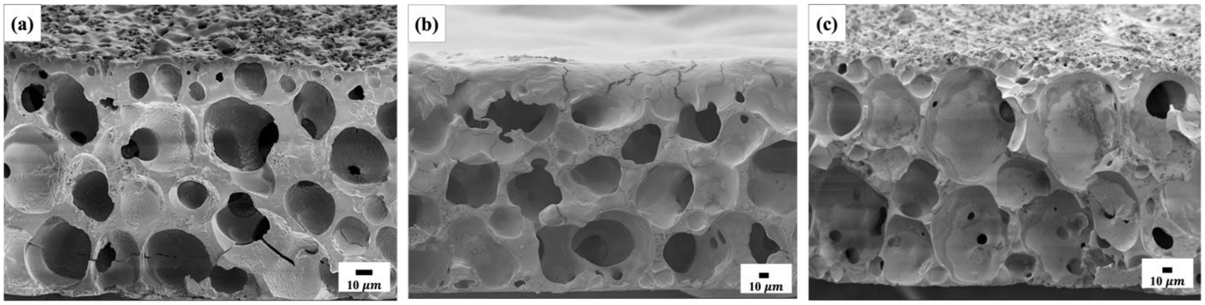

Figure S5. Low magnification cross-sectional SEM images of porous PIL membranes prepared with (a) 100 mg/ml, (b) 150 mg/ml and (c) 200 mg/ml amount of PIL dissolved in DMSO solution and TA as a phosphonic acid compound.

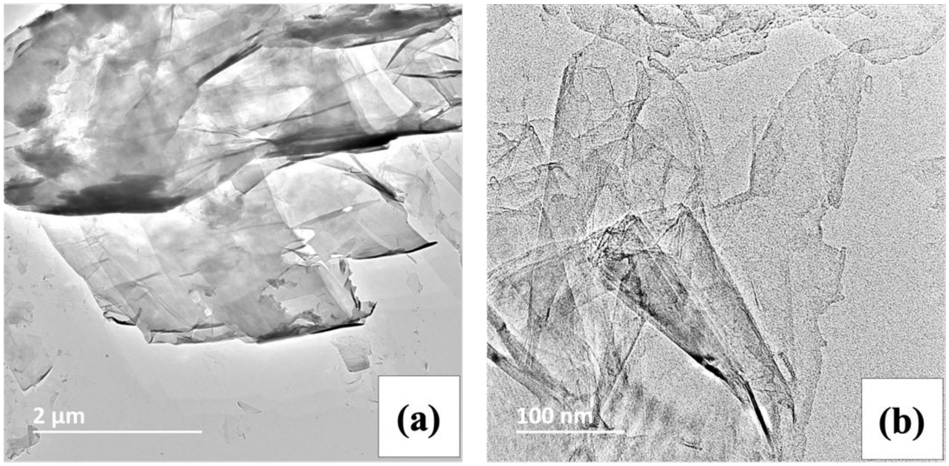

Figure S6. TEM images of MXene nanolayers with (a) low and (b) high magnification.

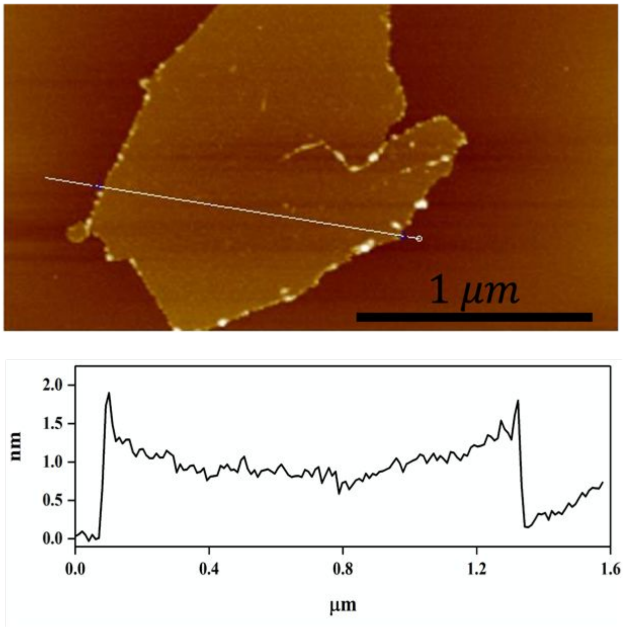

Figure S7. AFM image of MXene nanolayers with the thickness distribution.

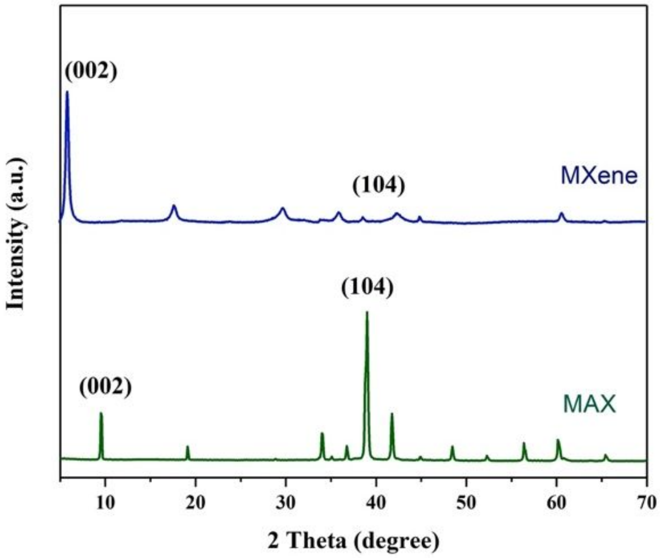

Figure S8. XRD patterns of MAX phase (Ti_3AlC_2) and MXene nanosheets ($Ti_3C_2T_x$).

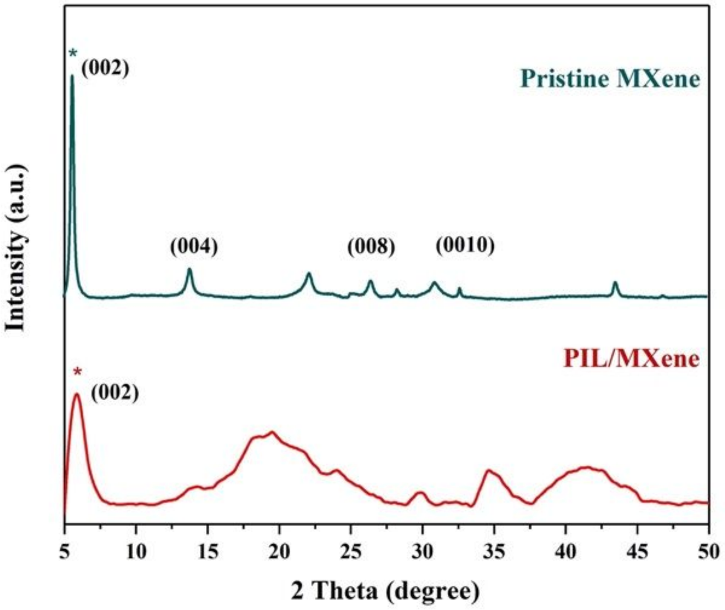

Figure S9. XRD patterns of pristine MXene and PIL/MXene dried membrane.

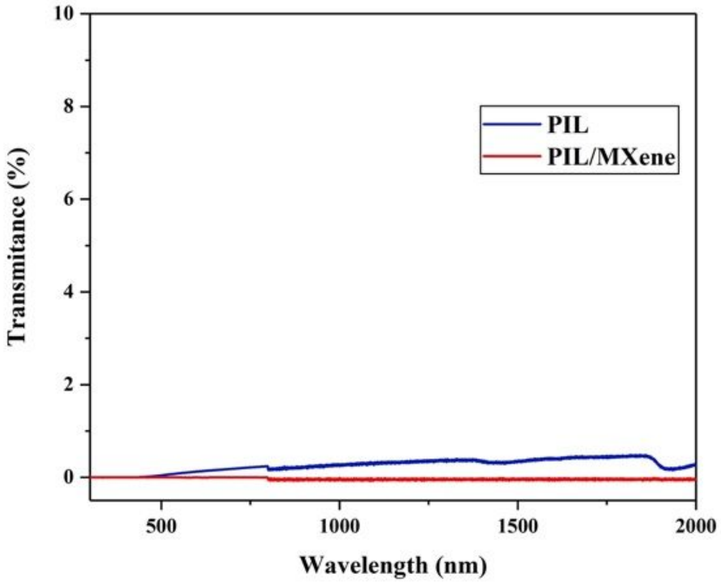

Figure S10. UV-Vis-NIR spectra of transmittance for PIL and PIL/MXene membranes.

Table S1. The comparison of evaporation rate and efficiency of this work with other composite materials.

Materials	Evaporation rate (Kg m^{-2} h^{-1})	Efficiency (%)	Ref.
Graphite film	1.01	62.7	[S1]

PPy-coated SS mesh	0.92	58	[S2]
F-wood/CNTs	0.95	65	[S3]
F-wood	1.05	72	[S4]
PPy–wood	1.014	72.5	[S5]
C-L-Wood	1.08	74	[S6]
CNT modified filter paper	1.15	75	[S7]
CNF/CNT aerogel	1.11	76.3	[S8]
Carbon Felt	1.22	79.4	[S9]
CNF membrane	1.32	82	[S10]
Carbonized mushroom	1.475	78	[S11]
Graphene oxide aerogels	1.395	83	[S12]
PDMS/CNTs@melamine	1.44	84	[S13]
carbon nanotube-loaded cotton fabric	1.18	86.01	[S14]
Co-NCNT-GO	1.63	88	[S15]
SiO_2/MXene/HPTFE	1.53	85.6	[S16]
Ag/PPy-PMBA-BrILs	1.37	88.7	[S17]
J-CNT/PVAs	1.34	85.7	[S18]
g-C_3N_4/MoS_2 and polyethylene foam	1.23	83.8 %	[S19]
Graphene based hive	1.63	85.0 %	[S20]
$W_{18}O_{49}$@PDMS	1.15	82.0 %	[S21]
Fe_2O_3/CNT/NF	1.23	81.3 %	[S22]
MXene/PVA/HA aerogel	0.72	61.0 %	[S23]
MXene/carbon nanotubes/cotton fabrics	1.35	88.2 %	[S24]
hydrophobic MXene	1.12	71.0 %	[S25]
MXene/cellulose	1.44	85.8 %	[S26]

MXene/PIL/PAA	1.45	95.0%	[S27]
**MXene/PIL**	**1.2**	**92.0%**	**This work**

References

[S1] Kashyap, V.; Al-Bayati, A.; Sajadi, S. M.; Irajizad, P.; Wang, S. H.; Ghasemi, H. A Flexible Anti-Clogging Graphite Film for Scalable Solar Desalination by Heat Localization. *J. Mater. Chem. A* **2017**, *5*, 15227-15234.

[S2] Zhang, L.; Tang, B.; Wu, J.; Li, R.; Wang, P.; Hydrophobic Light-to-Heat Conversion Membranes with Self-Healing Ability for Interfacial Solar Heating. *Adv. Mater.* **2015**, *27*, 4889-4894.

[S3] Chen, C.; Li, Y.; Song, J.; Yang, Z.; Kuang, Y.; Hitz, E.; Jia, C.; Gong, A.; Jiang, F.; Zhu, J. Highly Flexible and Efficient Solar Steam Generation Device. *Adv. Mater.* **2017**, *29*, 1701756.

[S4] Xue, G.; Liu, K.; Chen, Q.; Yang, P.; Li, J.; Ding, T.; Duan, J.; Qi, B.; Zhou, J. Robust and Low-Cost Flame-Treated Wood for High-Performance Solar Steam Generation. *ACS Appl. Mater. Interfaces* **2017**, *9*, 15052-15057.

[S5] Wang, Z.; Yan, Y.; Shen, X.; Jin, C.; Sun, Q.; Li, H. A Wood–Polypyrrole Composite as a Photothermal Conversion Device for Solar Evaporation Enhancement. *J. Mater. Chem. A* **2019**, *7*, 20706-20712.

[S6] Liu, H.; Chen, C.; Chen, G.; Kuang, Y.; Zhao, X.; Song, J.; Jia, C.; Xu, X.; Hitz, E.; Xie, H. High-Performance Solar Steam Device with Layered Channels: Artificial Tree with a Reversed Design. *Adv. Ener. Mater.* **2018**, *8*, 1701616.

[S7] Yang, P.; Liu, K.; Chen, Q.; Li, J.; Duan, J.; Xue, G.; Xu, Z.; Xie, W.; Zhou, J. Solar-Driven Simultaneous Steam Production and Electricity Generation from Salinity. *Ener. Environ. Sci.* **2017**, *10*, 1923-1927.

[S8] Jiang, F.; Liu, H.; Li, Y.; Kuang, Y.; Xu, X.; Chen, C.; Huang, H.; Jia, C.; Zhao, X.; Hitz, E. Lightweight, Mesoporous, and Highly Absorptive All-Nanofiber Aerogel for Efficient Solar Steam Generation. *ACS Appl. Mater. Interfaces* **2018**, *10*, 1104-1112.

[S9] Li, H.; He, Y.; Hu, Y.; Wang, X. Commercially Available Activated Carbon Fiber Felt Enables Efficient Solar Steam Generation. *ACS Appl. Mater. Interfaces* **2018**, *10*, 9362-9368.

[S10] Wang, Y.; Zhang, L.; Wang, P. Self-Floating Carbon Nanotube Membrane on Macroporous Silica Substrate for Highly Efficient Solar-Driven Interfacial Water Evaporation. *ACS Sustain. Chem. Engineer.* **2016**, *4*, 1223-1230.

[S11] Xu, N.; Hu, X.; Xu, W.; Li, X.; Zhou, L.; Zhu, S.; Zhu, J. Mushrooms as Efficient Solar Steam-Generation Devices. *Adv. Mater.* **2017**, *29*, 1606762.

[S12] Hu, X.; Xu, W.; Zhou, L.; Tan, Y.; Wang, Y.; Zhu, S.; Zhu, J. Tailoring Graphene Oxide-Based Aerogels for Efficient Solar Steam Generation under One Sun. *Adv. Mater.* **2017**, *29*, 1604031.

[S13] Li, Q.; Zhao, X.; Li, L.; Hu, T.; Yang, Y.; Zhang, J. Facile Preparation of

polydimethylsiloxane/Carbon Nanotubes Modified Melamine Solar Evaporators for Efficient Steam Generation and Desalination. *J. Colloid Interf. Sci.* **2021**, *584*, 602-609.

[S14] Qi, Q.; Wang, Y.; Wang, W.; Ding, X.; Yu, D. High-Efficiency Solar Evaporator Prepared by One-Step Carbon Nanotubes Loading on Cotton Fabric Toward Water Purification. *Sci. Total Environ.* **2020**, *698*, 134136.

[S15] Kong, Y.; Gao, Y.; Shang, Y.; Kong, W.; Qi, Y.; Wang, S.; Yin, F.; Gao, B.; Wang, S.; Yue, Q. Synergistic Adjustment of Water Channels and Light Absorption Pathways to Co-Generate Salt Collection and Clean Water Production. *Sci. Total Environ.* **2021**, *797*, 148912.

[S16] Li, H.; Li, L.; Xiong, L.; Wang, B.; Wang, G.; Ma, S.; Han, X. SiO_2/MXene/Poly(tetrafluoroethylene)-Based Janus Membranes as Solar Absorbers for Solar Steam Generation. *ACS Appl. Nano Mater.* **2021**, *4*, 14274-14284.

[S17] Xiao, C.; Liang, W.; Hasi, Q.-M.; Chen, L.; He, J.; Liu, F.; Wang, C.; Sun, H.; Zhu, Z.; Li, A. Ag/Polypyrrole Co-Modified Poly(ionic liquid)s Hydrogels as Efficient Solar Generators for Desalination. *Mater. Today Ener.* **2020**, *16*, 100417.

[S18] Jian, H.; Qi, Q.; Wang, W.; Yu, D. A Janus Porous Carbon Nanotubes/Poly(vinyl alcohol) Composite Evaporator for Efficient Solar-Driven Interfacial Water Evaporation. *Sep. Purif. Technol.* **2021**, *264*, 118459.

[S19] Gan, Q.; Xiao, Y.; Li, C.; Peng, H.; Zhang, T.; Ye, M. g-C_3N_4/MoS_2 Based Floating Solar Still for Clean Water Production by Thermal/Light Activation of Persulfate. *Chemosphere* **2021**, *280*, 130618.

[S20] Song, X.; Song, H.; Wang, S.; Liu, J.; Zhou, L.; Xu, J.; Chen, K. Enhancement of Solar Vapor Generation by a 3D Hierarchical Heat Trapping Structure. *J. Mater. Chem. A* **2019**, *7*, 26496-26503.

[S21] Chang, Y.; Wang, Z.; Shi, Y.-e.; Ma, X.; Ma, L.; Zhang, Y.; Zhan, J. Hydrophobic $W_{18}O_{49}$ Mesocrystal on Hydrophilic PTFE Membrane as an Efficient Solar Steam Generation Device Under One Sun. *J. Mater. Chem. A* **2018**, *6*, 10939-10946.

[S22] Han, S.; Yang, J.; Li, X.; Li, W.; Zhang, X.; Koratkar, N.; Yu, Z.-Z. Flame Synthesis of Superhydrophilic Carbon Nanotubes/Ni Foam Decorated with Fe2O3 Nanoparticles for Water Purification via Solar Steam Generation. *ACS Appl. Mater. Interfaces* **2020**, *12*, 13229-13238.

[S23] Cao, S.; Wu. X.; Zhu, Y.; Gupta, P.; Martinez, A.; Zhang, Y.; Ghim, D.; Wang, Y.; Liu, L.; Jun, Y.-S.; Singamaneni, S. MXene Aerogel for Efficient Photothermally Driven Membrane Distillation with Dual-Mode Antimicrobial Capability. *J. Mater. Chem. A* **2021**, *9*, 22585-22596.

[S24] Wang, Y.; Qi, Q.; Fan, J.; Wang, W.; Yu, D. Simple and Robust MXene/Carbon

Nanotubes/Cotton Fabrics for Textile Wastewater Purification via Solar-Driven Interfacial Water Evaporation. *Sep. Purif. Technol.* **2021**, *254*, 117615.

[S25] Zhao, J.; Yang, Y.; Yang, C.; Tian, Y.; Han, Y.; Liu, J.; Yin, X.; Que, W. A Hydrophobic Surface Enabled Salt-Blocking 2D Ti_3C_2 MXene Membrane for Efficient and Stable Solar Desalination. *J. Mater. Chem. A* **2018**, *6*, 16196-16204.

[S26] Zha, X.-J.; Zhao, X.; Pu, J.-H. ; Tang, L.-S.; Ke, K.; Bao, R.-Y.; L. Bai, L.; Liu, Z.-Y.; Yang, M.-B.; Yang, W. Flexible Anti-Biofouling MXene/Cellulose Fibrous Membrane for Sustainable Solar-Driven Water Purification. *ACS Appl. Mater. Interfaces* **2019**, *11*, 36589-36597.

[S27] Kheirabad, A.K.; Chang, J.; Zhang, M.; and Yuan, J. MXene/Poly(ionic liquid) Porous Composite Membranes for Systematized Solar-Driven Interfacial Steam Generation. *2D Materials*, **2023**, *10*(2), 024008.
